# EGFR-Targeted Hybrid Plasmonic Magnetic Nanoparticles Synergistically Induce Autophagy and Apoptosis in Non-Small Cell Lung Cancer Cells

**DOI:** 10.1371/journal.pone.0025507

**Published:** 2011-11-07

**Authors:** Tomohisa Yokoyama, Justina Tam, Shinji Kuroda, Ailing W. Scott, Jesse Aaron, Tim Larson, Manish Shanker, Arlene M. Correa, Seiji Kondo, Jack A. Roth, Konstantin Sokolov, Rajagopal Ramesh

**Affiliations:** 1 Department of Thoracic and Cardiovascular Surgery, The University of Texas M. D. Anderson Cancer Center, Houston, Texas, United States of America; 2 Department of Biomedical Engineering, The University of Texas at Austin, Austin, Texas, United States of America; 3 Department of Neurosurgery, The University of Texas M. D. Anderson Cancer Center, Houston, Texas, United States of America; 4 Department of Imaging Physics, The University of Texas M. D. Anderson Cancer Center, Houston, Texas, United States of America; 5 Department of Pathology, University of Oklahoma Health Sciences Center, Oklahoma City, Oklahoma, United States of America; 6 Graduate Program in Biomedical Sciences, University of Oklahoma Health Sciences Center, Oklahoma City, Oklahoma, United States of America; Ohio State University, United States of America

## Abstract

**Background:**

The epidermal growth factor receptor (EGFR) is overexpressed in 80% of non-small cell lung cancer (NSCLC) and is associated with poor survival. In recent years, EGFR-targeted inhibitors have been tested in the clinic for NSCLC. Despite the emergence of novel therapeutics and their application in cancer therapy, the overall survival rate of lung cancer patients remains 15%. To develop more effective therapies for lung cancer we have combined the anti-EGFR antibody (Clone 225) as a molecular therapeutic with hybrid plasmonic magnetic nanoparticles (NP) and tested on non-small cell lung cancer (NSCLC) cells.

**Methodology/Principal Findings:**

Cell viability was determined by trypan-blue assay. Cellular protein expression was determined by Western blotting. C225-NPs were detected by electron microscopy and confocal microscopy, and EGFR expression using immunocytochemistry. C225-NP exhibited a strong and selective antitumor effect on EGFR-expressing NSCLC cells by inhibiting EGFR-mediated signal transduction and induced autophagy and apoptosis in tumor cells. Optical images showed specificity of interactions between C225-NP and EGFR-expressing NSCLC cells. No binding of C225-NP was observed for EGFR-null NSCLC cells. C225-NP exhibited higher efficiency in induction of cell killing in comparison with the same amount of free C225 antibody in tumor cells with different levels of EGFR expression. Furthermore, in contrast to C225-NP, free C225 antibody did not induce autophagy in cells. However, the therapeutic efficacy of C225-NP gradually approached the level of free antibodies as the amount of C225 antibody conjugated per nanoparticle was decreased. Finally, attaching C225 to NP was important for producing the enhanced tumor cell killing as addition of mixture of free C225 and NP did not demonstrate the same degree of cell killing activity.

**Conclusions/Significance:**

We demonstrated for the first time the molecular mechanism of C225-NP induced cytotoxic effects in lung cancer cells that are not characteristic for free molecular therapeutics thus increasing efficacy of therapy against NSCLC.

## Introduction

The epidermal growth factor receptor (EGFR) is overexpressed in 80% of NSCLC and is associated with poor survival [1]. In recent years, EGFR-targeted inhibitors have been tested in the clinic for NSCLC [2]–[6]. However, the emerging molecular therapeutics has produced modest increases in patient survival over survival of patients receiving standard non-targeted treatments. As a result the overall survival rate of lung cancer patients remains less than 16% [7]. Thus, there is continued stimulus to develop and test novel therapies. Our approach to overcome the limitation of current therapies has been to combine molecular therapeutics with the emerging field of nanotechnology and test against lung cancer.

It has been convincingly shown that nanotechnology can provide unique solutions to revolutionize diagnosis and treatment of many devastating diseases such as cancer [1], [8]–[11]. One specific area of great interest is development of nanoparticles for molecular specific imaging, therapy and combined imaging/therapy [12]–[13]. Multiplexing different types of nanoparticles (NPs) and targeting molecules provides a common platform for multiple imaging applications with a high degree of flexibility [14]–[15]. Although imaging and photothermal therapy with plasmonic and hybrid plasmonic NPs have been quite extensively studied, the molecular mechanisms of NP interactions with live cells is not fully understood. Recently, it was demonstrated that NPs interact with cells in a size-dependant manner with 40–50 nm NPs showing the greatest cellular uptake [16]. However, in this study the molecular signaling effects following NPs uptake was not investigated.

In the present study we combined the anti-EGFR antibody (Clone 225) as a molecular therapeutic with hybrid plasmonic magnetic NPs and studied the molecular interactions between EGFR-targeted NP (225-NP) in the size range of 40–50 nm and human non-small cell lung cancer (NSCLC) cells. The 225-NP consists of a paramagnetic iron core that is surrounded by a gold layer and is functionalized with monoclonal anti-EGFR antibodies (Clone 225). We used human lung cancer cells with different levels of EGFR expression and changed the surface composition of NP to elucidate mechanisms of these interactions. Our initial goal was to use C225-NP as an imaging agent targeted to EGFR overexpressing lung cancer cells. Unexpectedly, we found that the C225-NP was selectively cytotoxic for EGFR-overexpressing lung cancer cells beyond what would be expected from the unconjugated antibody. Our results provide a new direction toward increasing potency of molecular specific therapeutics through their three-dimensional arrangement on the nanoscale using NPs as templates.

## Results and Discussion

### 225-NP-treatment regulates EFGR-signaling pathway and kill's lung tumor cells more effectively than normal cells

**Figure 1:**
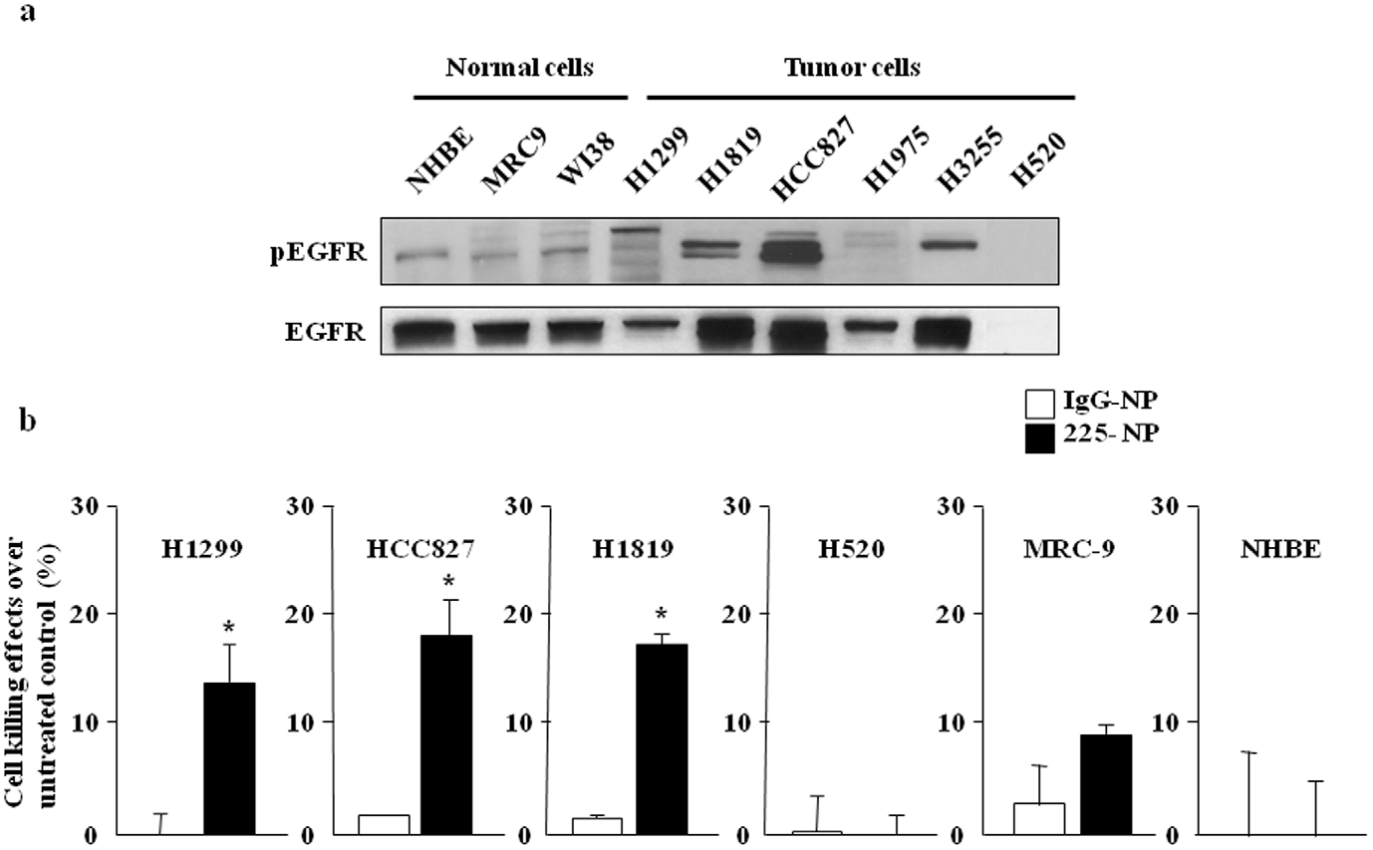
Effect of EGFR-targeted C225-NP treatment on NSCLC cells. (**a**) Expression of phosphorylated and total EGFR in normal and NSCLC cells. (**b**) Cell killing in response to EGFR-targeted C225-NP on NSCLC and normal cells. Results shown are the means ± S.D. of three independent experiments. *P-value<0.05 vs IgG-NP on H1299, HCC827, and H1819 cells.

First, we examined EGFR (phosphorylated and total) expression in several human NSCLC cell lines that are wild type (H1229), mutated and overexpressed (HCC827, H1975, H3255), amplified (H1819), or null (H520) for EGFR and compared with EGFR expression in normal lung fibroblasts (MRC-9 and WI38) and normal human bronchial epithelial cells (NHBE) by Western blotting. Both total and phosphorylated EGFR (pEGFR) expression was detected in all cell lines tested except for H520 cells which are null for EGFR. However, the pEGFR expression levels varied among the cell lines with high expression detected in HCC827, H1819 and H3255 cells (**Figure 1a**). H1299 cells had an intermediate expression level of pEGFR. In contrast, normal cells (NHBE, MRC-9, and WI38) and H1975 cells expressed low levels of pEGFR. Treatment with C225-NP significantly (P = <0.05) decreased the viability of EGFR positive HCC827, H1299, and H1819 cancer cells compared to treatment with control IgG-NP (**Fig. 1b**). The C225-NP-mediated inhibitory effects however varied among the cancer cell lines tested and was independent of EGFR mutational status. No inhibitory effects were observed in C225-NP-treated H520 cells when compared to IgG-NP-treated cells. Among the normal cells, MRC-9 but not NHBE cells showed some inhibitory effects when treated with C225-NP and compared with IgG-NP treatment (**Figure 1b**). However, the inhibitory effects observed in C225-NP-treated MRC-9 (9%) cells were less than the inhibitory effects observed in C225-NP-treated tumor cells (14–18%).

**Figure 2:**
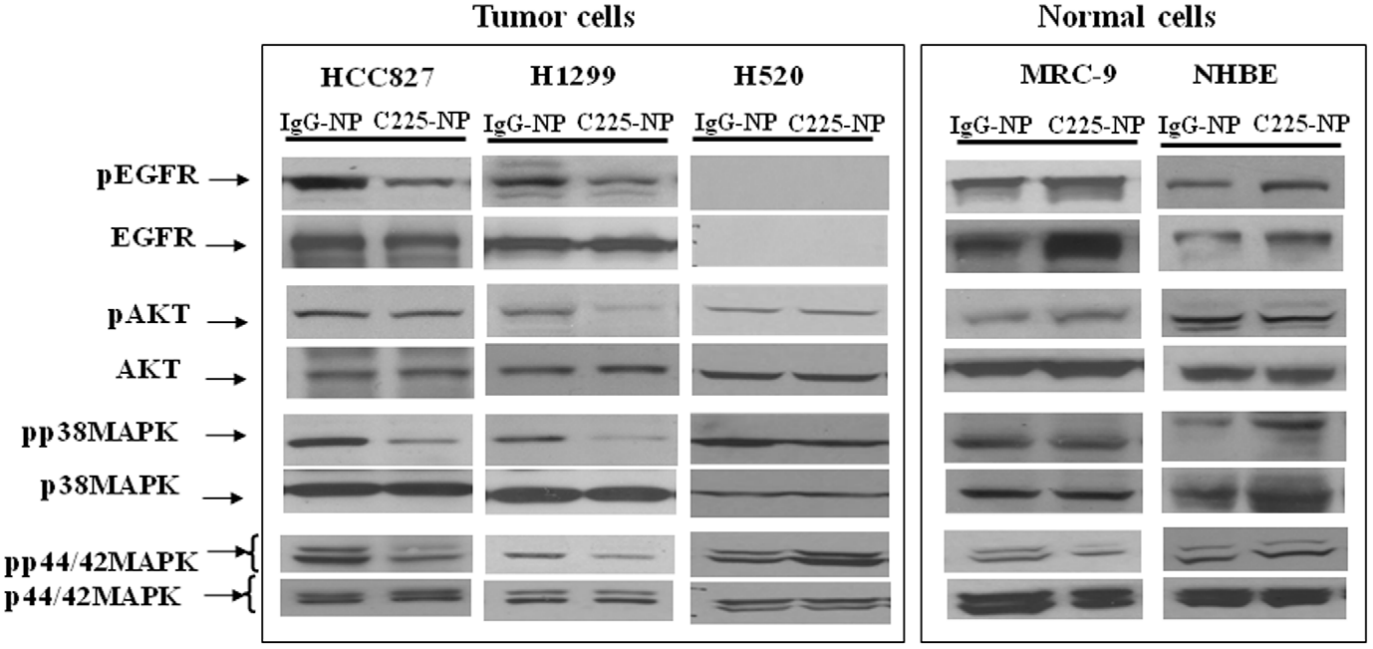
C225-NP regulates EGFR signaling pathway in tumor cells but not in normal cells. Inhibitory effects of C225-NP on phosphorylated EGFR and the downstream signaling molecules in human lung cancer and normal cells. C225-NP treatment reduced the expression of phosphorylated (p) forms of EGFR, AKT, p38 MAPK, and P44/42 MAPK in EGFR-positive tumor cells but not in normal cells. There was no effect on EGFR-null H520 tumor cells.

Then, we assessed the protein expression of phosphorylated EGFR and EGFR-associated signaling molecules in tumor and normal cells after treatment with the IgG- or C225-NP. Reduced expression of phosphorylated-EGFR, -AKT, -p38MAPK, and p44/42MAPK after C225-NP treatment was observed in HCC827 and H1299 cells when compared to cells treated with control IgG-NP (**Figure 2**). In H520 cells the expression of all of these proteins remained unchanged when treated with 225-NP and compared with cells treated with IgG-NP (**Figure 2**). Immunohistochemical analysis showed pEGFR expression was markedly reduced (35%; *P*<0.05) in HCC827 cells at 30 and 60 minutes after treatment with C225-NP compared to pEGFR expression in IgG-NP-treated cells (8–12%; **Figure S1**). In normal (MRC-9 and NHBE) cells, there was no marked reduction in pEGFR or the associated proteins analyzed from IgG-NP- and C225-NP-treatment (**Figure 2**).

### 225-NP-treatment produces greater antitumor activity than C225 and NP treatment alone

**Figure 3:**
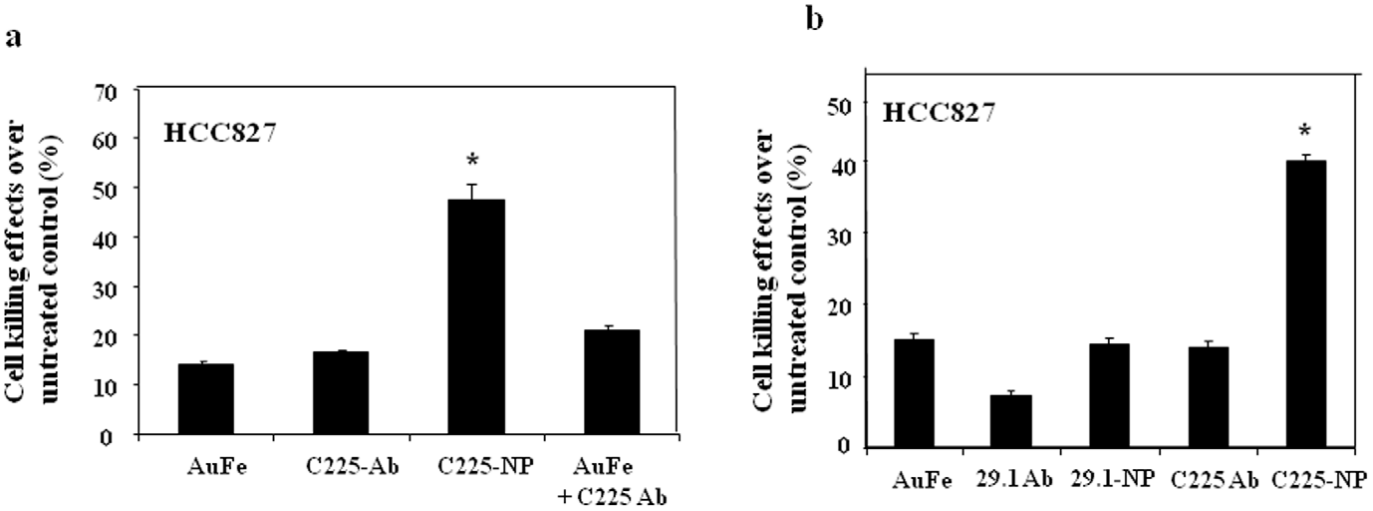
C225-NP produces a greater killing effect than individual components of C225-NP. (**a**) HCC827 cells treated with C225-NP demonstrated significant tumor killing effect compared to treatment with AuFe, 225 antibody and AuFe plus C225 antibody. * P value is <0.05. (**b**) Comparison of the inhibitory effects produced by 29.1-NP with C225-NP on HCC827 cells. C225-NP treatment produced a greater killing effect than did 29.1-NP treatment compared to other treatment groups. * P value is <0.05.

Next, we determined the contribution of the individual components constituting the C225-NP on the observed growth inhibitory effects in tumor cells. HCC827 tumor cells treated with AuFe alone, and free C225-antibody alone reduced cell viability by 12–14% over untreated control cells while combination treatment with free C225-antibody plus AuFe reduced cell numbers by 22% (**Figure 3a**). Intriguingly, C225-NP greatly reduced the cell number (48%; P<0.05) that was markedly higher than the inhibitory effect produced by individual constituents (225 antibody, AuFe) indicating conjugation of C225 antibodies to NP enhances the anticancer activity of C225-NPs. To further validate our findings we compared the inhibitory effects of C225 antibody with Clone 29.1 antibody. Both Clone 225 and Clone 29.1 antibodies bind to EGFR. However, Clone 29.1 unlike Clone 225 binds to a carbohydrate residue on the external portion of EGFR and does not inhibit EGF-mediated EGFR-signaling [17]. Treatment of HCC827 cells with C225-NP produced the greatest inhibitory effect (∼42%; P<0.05; **Figure 3b**) that was significantly higher when compared to the inhibitory effect produced by Clone 29.1-NP (∼14%) and other control treatments (∼7%–15%). These results demonstrate conjugation of Clone 225 antibodies to AuFe NP confers a unique property that enhances the antitumor activity against EGFR- positive cancer cells. Similar to our findings, conjugation of anti-ErbB2 antibody to spherical gold NP demonstrated increased killing of breast cancer cells as compared to anti-ErbB2 antibody and NP alone [18]. In this study the cytotoxicity was investigated as a function of NP size. However, the study did not address the molecular mechanism for enhanced tumor cell killing. The importance of surface density of antibodies attached to nanoparticles also was not analyzed.

### 225-NP-treatment induces autophagy and apoptosis in EGFR-positive tumor cells but not in EGFR-negative tumor cells

**Figure 4:**
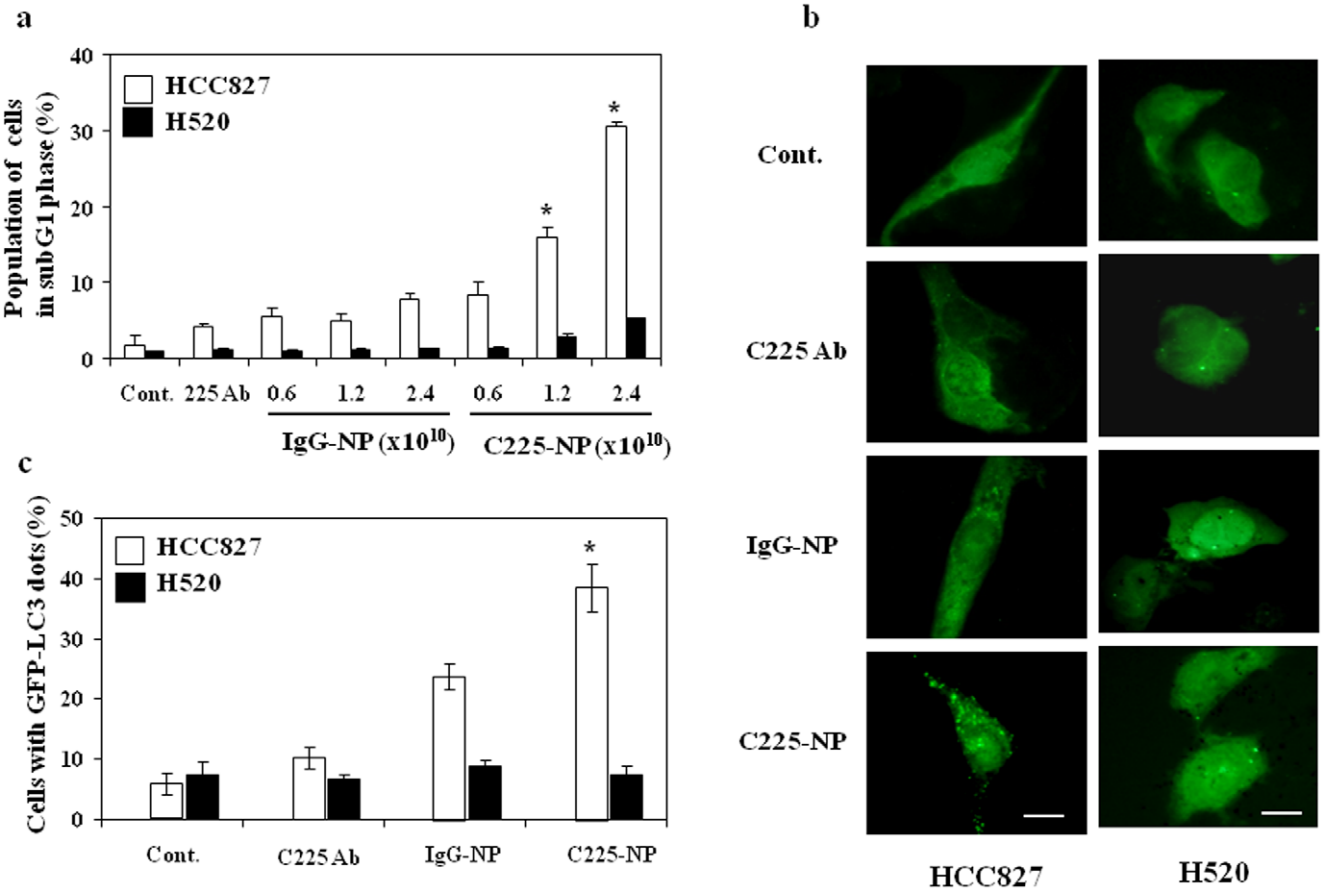
Induction of apoptosis and autophagy in response to EGFR-targeted C225-NP in NSCLC cells. (**a**) Percentage of NSCLC cells treated with different doses (0.6, 1.2 and 2.4×10^10^ particles) of IgG-NP or C225-NP for 72 hrs that was in the sub-G_1_ phase of the cell cycle. This percentage was determined by DNA flow-cytometric analysis. Untreated cells and cells treated with C225 antibody alone served as control. A dose-dependent increase in the number of HCC827 cells in subG1 phase was observed in both IgG-NP and C225-NP treatments. However, the increase in number of cells in subG1 was significantly higher in C225-NP treatment than in IgG-NP (^*^P<0.05). There was no marked increase in subG1 phase in H520 cells between IgG-NP- and C225-NP-treatments. (**b**) Detection of GFP-LC3 dots indicative of autophagy on NSCLC cells that were not treated or treated with C225 antibody, IgG-NP or C225-NP (3×10^9^ particles) for 72 hrs on chamber slides. *Scale bar = 50 µm* (**c**) Quantification of the number of cells with GFP-LC3 dots on untreated and treated NSCLC cells. The number of cells with GFP-LC3 dots was higher in C225-NP-treated HCC827 cells compared to all other treatment groups. In H520 cells there was no increase in the number of GFP-LC3 dots when treated with C225-NP and compared to all other treatment groups. Results shown are the means ± S.D. of three independent experiments. *P-value <0.05 vs untreated control, C225 antibody, and IgG-NP on HCC827 cells.

Here to determine the molecular mechanism of C225-NP-mediated tumor growth inhibition we performed flow cytometric analyses on C225-NP-treated HCC827 and -H520 cells. As shown in **Figure 4a** **,** the sub-G1 population, which indicates the percentage of apoptotic cells, increased in a dose-dependent manner after treatment with C225-NP (9%–20%) on HCC827 cells compared to control IgG-NP-treated cells (4%–8%). C225-NP treatment markedly increased the number of cells in sub-G1 population than did treatment with Clone 225 antibody alone at equimolar concentration (**Figure S2**). As expected, there was no increase in the number of cells in sub-G1 population in C225-NP-treated H520 cells compared to other treatments (**Figure 4a**). Thus, C225-NP effectively induced apoptosis on EGFR-expressing HCC827 cells but not in EGFR null H520 cells.

Recently, it has been shown that quantum dots induce size-dependent autophagy in human mesenchymal stem cells [19]. We therefore examined whether autophagy occurred in NSCLC cells after treatment with C225-NP. The green fluorescent protein (GFP)-tagged expression vector of LC3 is a useful tool for detecting autophagy [20]. On fluorescent microscopy, GFP-LC3-transfected HCC827 cells showed diffuse distribution of GFP-LC3 for untreated cells, and cells treated with Clone 225 antibody alone and with IgG-NP, whereas the cells treated with C225-NP showed GFP-LC3 punctate dots indicative of autophagic vacuoles (**Figure 4b**). The percentage of cells with GFP-LC3 punctate dots increased after treatment with C225-NP for HCC827 cells (**Figure 4c** **;** P<0.05). Induction of autophagy determined by GFP-LC-3 dots was also markedly increased in C225-NP-treated H1299 cells compared to other treatment groups (**Figure S3**). In contrast, the amount of GFP-LC3 punctate dots was not different in C225-NP treated H520 cells and control groups (**Figure 4b, c**).

**Figure 5:**
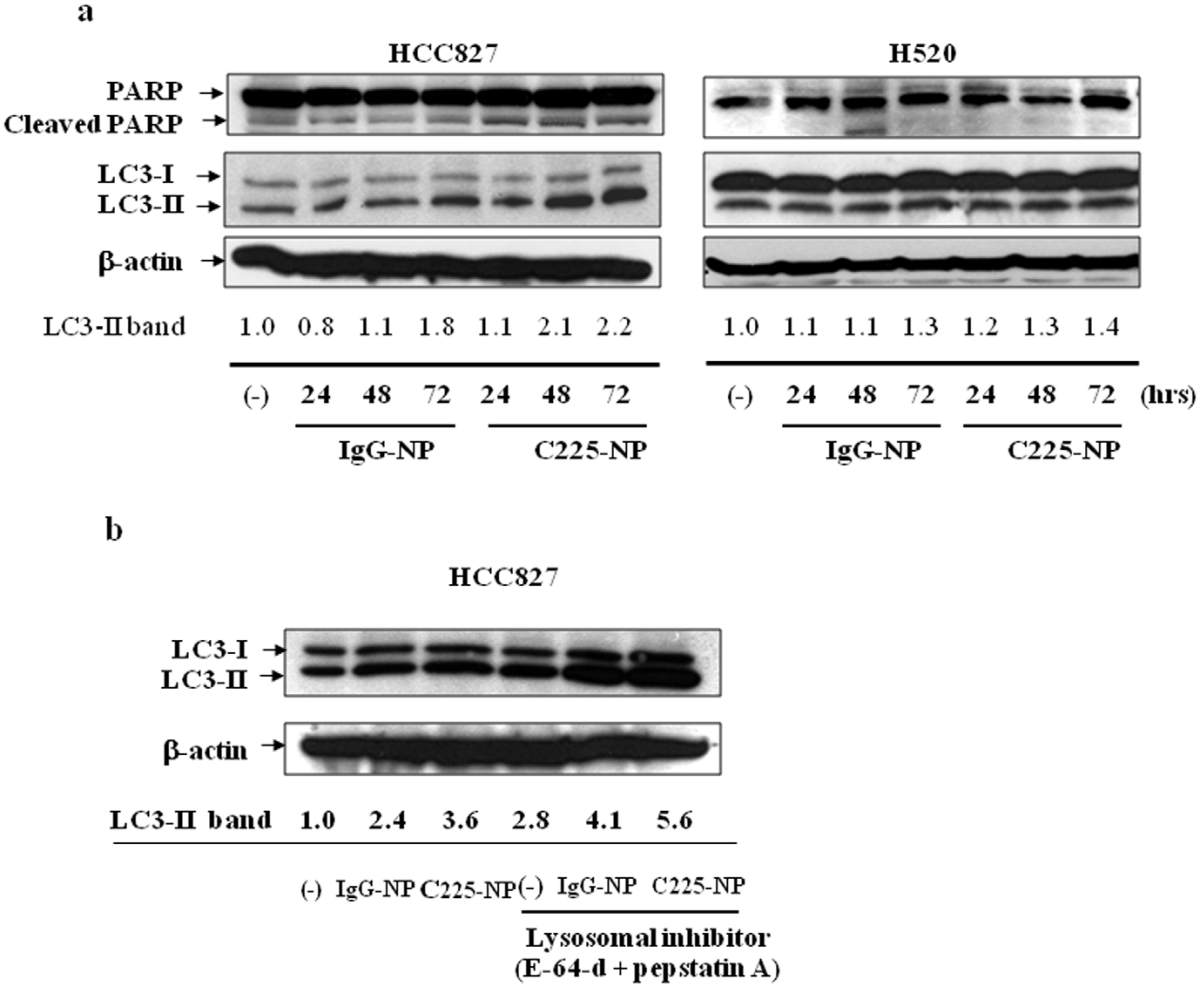
Analysis for molecular markers of autophagy and apoptosis in C225-NP-treated cancer cells. (**a**) Cellular proteins were lysed at the indicated time after treatment with IgG-NP particles or C225-NP (0.6×10^10^ particles) and subjected to Western blotting. In HCC827 but not in H520 cells, increased PARP cleavage and LC3-II was observed at 48 h and 72 h after treatment with C225-NP and when compared with IgG-NP treatment and untreated control. The intensities of the amount of LC3-II bands were semi-quantified by ImageJ software (National Institutes of Health, Bethesda, MD) (**b**) HCC827 cells were treated with IgG-NP or C225-NP for 68 hrs, cells were further cultured with or without protease inhibitors [10 µg/ml E-64-d and 10 µg/ml pepstatin A (BIOMOL International L.P., Plymouth Meeting, PA)] for 4 hrs. Cellular proteins were lysed and immunoblotted with anti-LC3 antibody. LC3-II protein levels were increased in the presence of protease inhibitors in all of the groups indicating occurrence of autophagy. The intensities of the amount of LC3-II bands were semi-quantified by ImageJ software (National Institutes of Health).

We next examined the cells treated with C225-NP for the presence and timing of poly (ADP-ribose) polymerase (PARP) cleavage and LC3 expression, which are molecular markers indicative of cells undergoing apoptosis and autophagy respectively. PARP cleavage was evident at 24 hrs after treatment with IgG-NP- and C225-NP of HCC827, but not H520 cells; however the PARP cleavage was higher with C225-NP than with control-NP in HCC827 cells (**Figure 5a**). The LC3 protein exists in two cellular forms, LC3-I and LC3-II. LC3-I is converted to LC3-II by conjugation to phosphatidylethanolamine, and the amount of LC3-II is closely correlated with the number of autophagosomes [21]. In HCC827 cells, treatment with C225-NP greatly increased the amount of LC3-II in a time dependent manner (**Figure 5a**). In addition, recent evidence suggests that the accumulation of LC3-II is more accurately represented in autophagic flux in the presence of lysosomal inhibitors [22]. In the presence of the protease inhibitors E-64-d and pepstatin A, endogenous LC3-II increased after treatment with IgG-NP and C225-NP in HCC827 cells (**Figure 5b**). However, the increase in LC3-II was greater in cells treated with C225-NP than IgG-NP. Increase in LC3-II in the presence of protease inhibitors was also observed in C225-NP-treated H1299 cells (data not shown). In contrast, the amount of LC3-II was not increased in H520 cells during 72 hrs treatment with C225-NP compared to treatment with IgG-NP (**Figure 5a**). These results indicated that C225-NP caused both apoptosis and autophagy at the same time in EGFR-expressed NSCLC cells. Surprisingly, autophagy was also observed in IgG-NP-treated cells albeit less than that observed in C225-NP-treated cells and had less effect on tumor cell killing. We hypothesize that the autophagy threshold in C225-NP-treated cells is stronger due to the presence of anti-EGFR antibodies and acts as a death activator leading to activation of the caspase cascade and apoptotic cell death. In contrast, the autophagy in IgG-NP-treated cells likely serves as a survival signal and protects from cell death. Additional differences may exist and we are currently investigating the mechanism in the laboratory.

**Figure 6:**
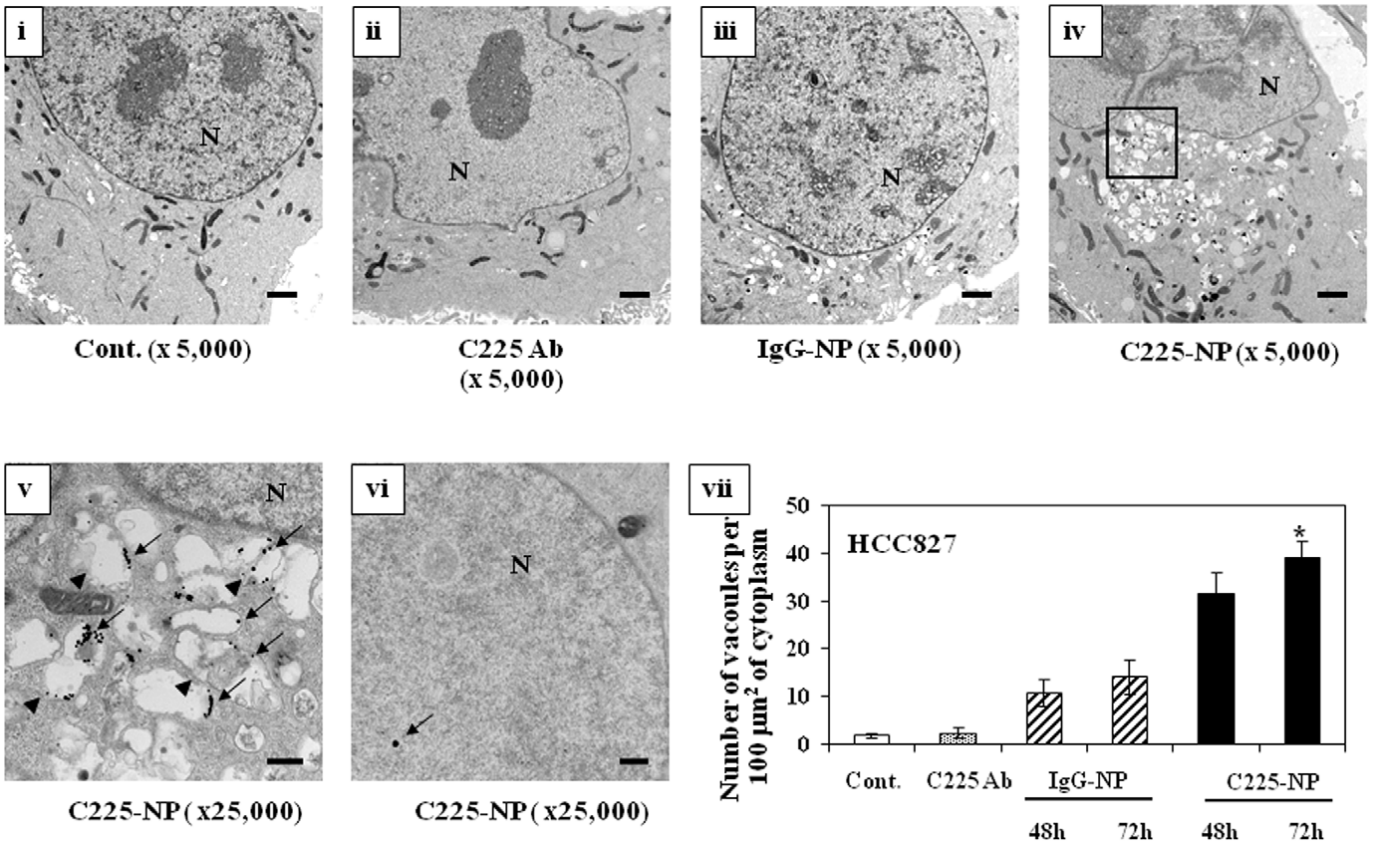
*In situ* localization of C225-NP in NSCLC cells. Electron photomicrographs showing the ultrastructure of a cell, including the nucleus (N), of HCC827 cells treated with C225 antibody (0.065 µg/ml), IgG-NP, or C225-NP (0.6×10^10^ particles) for 72 hrs. (**i**) Untreated cells (**ii**) C225 antibody-treated cells (**iii**) IgG-NP-treated cells (**iv**) C225-NP-treated cells (**v**) A magnified view of the area boxed in (**iv**). The arrow indicates NP, and the arrowhead indicates autophagosomes that includes residual material and NP in the cytoplasm (**vi**) C225-NP detected inside the nucleus. The arrow indicates NP in the nucleus. *Scale bar = 1 µm.* (**vii**) Autophagosomes were quantified, as described in Materials and Methods. *P-value <0.05 vs control, C225 antibody and IgG-NP for 48 and 72 hrs.

**Figure 7:**
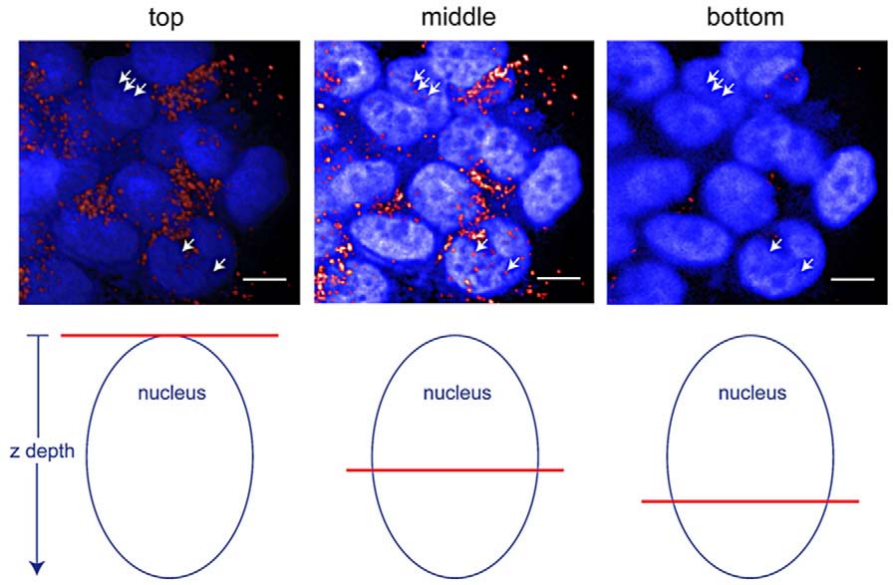
Detection of C225-NP localization in the nucleus by confocal microscopy. (Top row) Confocal images show DAPI stained nuclei (blue) and C225-NP (red). Arrows in the “middle” column point to C225-NP localized in a nucleus. Arrows in the columns labeled “top” and “bottom” point to the same area in the xy plane of the nucleus but at locations above and below the middle of the nucleus, respectively. In both the “top” and “bottom” images, the arrows are no longer pointing at red spots which indicate that the red spots in the “middle” image are indeed within the nucleus. (Bottom row) Cartoon indicating the z position of each image within the nucleus, where the red horizontal line represents the relative depth position within the nucleus that the cross sectional images (top row) were taken at. *Scale bar = 10 µm*.

To analyze the association between C225-NP and autophagy, we determined the localization of C225-NP and autophagic vacuoles, using transmission electron microscopy (TEM). HCC827 cells treated with Clone 225 antibody or IgG-NP exhibited very few autophagic features, whereas many autophagic vacuoles were observed after treatment with C225-NP (**Figure 6** **;** ***P*** **<0.05**). Interestingly, while most of the C225-NPs were observed inside autophagic and empty vacuoles, some NPs were also observed inside the nuclei. Similar to TEM analysis, confocal microscopy also revealed some NPs inside nuclei in C225-NP-treated cells (**Figure 7**). Although we could demonstrate presence of NPs in the nucleus of the cells we did not quantitate the number of NPs present inside the nucleus of the cell. This is because of some of the limitations that exist with TEM and confocal microscopy techniques. For example, the quantitation of the percentage of nuclear localization would require analyses of multiple cells through total cellular volume that is impractical using such high resolution technique as TEM. Three dimensional localization of NPs with confocal microscopy is complicated by the fact that after cellular uptake the NPs accumulate in the perinuclear space. Therefore, optical resolution is not sufficient to separate NPs which are located in the close proximity of the nuclear membrane on the cytoplasmic and on the nuclear sides. In addition, confocal microscopy from very bright objects such as NPs can result in a strong out of focus signal that can lead to misinterpretation of three-dimensional distribution of the nanoparticles. The limitations of three-dimensional localization of NPs using confocal optical microscopy have been recently demonstrated in [23] where confocal microscopy grossly overestimated the cytosolic uptake of quantum dots.

In future studies we plan to quantify the percentage of NPs present in the nucleus by three-dimensional X-ray tomographic imaging of whole cells with sufficient sampling size and resolution [24] and determine the contribution of nuclear NPs in cell signaling events.

### C225-NP is specific for EGFR-expressing tumor cells and pretreatment with C225 antibody abrogates C225-NP-mediated tumor cell killing

**Figure 8:**
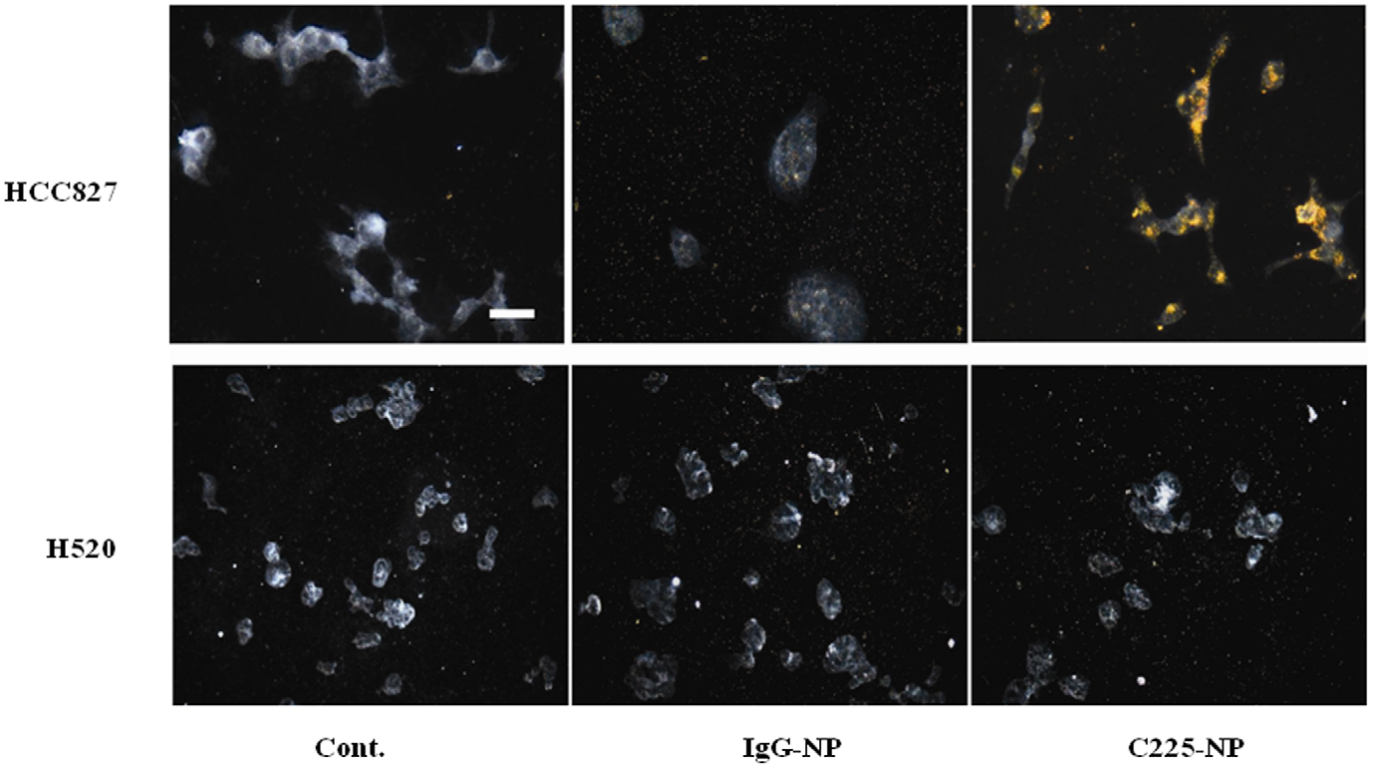
Visualization, and determination of selective binding and uptake of C225-NP in NSCLC cells. HCC827 and H520 cells seeded on chamber slides were treated with IgG-NP or C225- NP (0.2×10^10^ particles). Untreated cells served as control. At 24 hrs after treatment cells were washed, fixed and images were taken under dark-field microscopy. Scale bar is 50 micron. Selective binding and uptake of C225-NP but not IgG-NP was observed in HCC827 cells. In H520 cells there was no binding and uptake of C225-NP when compared with IgG-NP.

We used dark-field reflectance microscopy to characterize the specificity of C225-NP interactions and the feasibility of the optical detection of lung cancer cells. Dark-field reflectance images showed high concentration and internalization of C225-NP in HCC827 cells following 24 hour treatment (**Figure 8**). In contrast, little-to-no uptake was observed in HCC827 cells treated with IgG-NP. In H520 cells, no difference in NP binding and uptake was observed between cells treated with C225-NP and IgG-NP (**Figure 8**).

**Figure 9:**
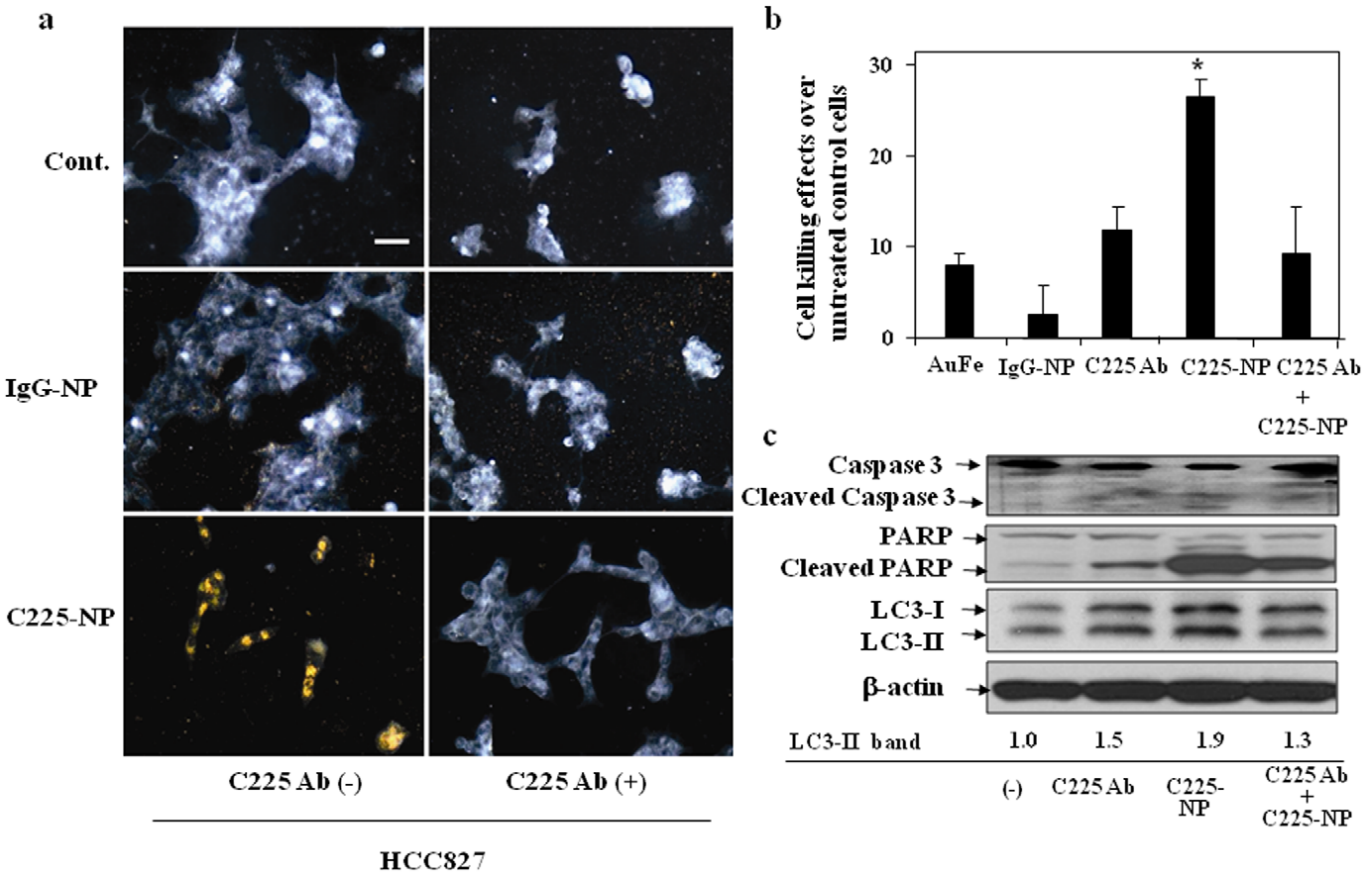
C225-NP is specific for EGFR-expressing tumor cells and pretreatment with C225 antibody abrogates C225-NP-mediated tumor cell killing. (**a**) The right column shows images of HCC827 cells pre-treated with C225 antibody (2 µg/ml) for 15 min before incubation with either IgG-NP (middle row) or C225-NP (bottom row). Cells shown in the left column were not treated with free C225 antibodies. The slides were washed, fixed and imaged using dark-field microscopy. Binding and uptake of C225-NP was completely inhibited in the presence of C225 antibody. In IgG-NP-treated cells C225 antibody had no effect. Scale bar is 50 micron. (**b**) Inhibition effect on the cytotoxicity of C225-NP by pre-treatment with free C225 antibody. After treatment with/without C225 antibody (0.065 µg/ml) for 6 hrs, the cells were treated with either non-conjugated NP (gold-iron: AuFe), IgG-NP or C225-NP for additional 66 hrs. The number of viable cells was counted as described in Materials and Methods. Results shown are the means ± S.D. of three independent experiments. *P-value <0.05 vs AuFe alone, C225 antibody alone, IgG-NP or C225 antibody plus C225-NP. (**c**) Inhibition effects on C225-NP-induced apoptosis and autophagy by pre-treatment with C225 antibody. Cellular proteins were lysed after treatment with C225-NP (0.6×10^10^ particles) for 66 hrs in the presence or absence of free C225 antibody (0.065 µg/ml). Proteins were separated by 7.5% or 15% SDS-PAGE, and immunoblotted with anti-PARP and anti-LC3 antibodies. The anti-β-actin antibody was used as a control for protein loading and transfer. The intensities of the amount of LC3-II bands were quantified by ImageJ software (National Institutes of Health). C225-NP-mediated activation of apoptosis and autophagy as indicated by cleavage of capase-3, PARP and LC3-II respectively was markedly abrogated in the presence of free C225 antibody. All dark-field images were acquired using Leica, DM6000 microscope equipped with 20× dark-field objective and Xe-lamp white light illumination.

To determine the specificity of the C225-NP binding and uptake, blocking experiments were conducted. HCC827 cells were pre-treated with excess of free Clone 225 antibody prior to treatment with IgG-NP or C225-NP. Dark-field reflectance images showed that binding and internalization of C225-NP was specifically blocked in the presence of free Clone 225 antibody as compared to cells that were not pretreated with the antibody (**Figure 9a**). We further determined whether the blocking of EGF receptor affects C225-NP-induced cytotoxicity. Attenuation of cytotoxicity mediated by C225-NP was observed in the presence of free Clone 225 antibody that was accompanied by marked reduction in C225-NP-mediated apoptosis and autophagy (**Figure 9b, 9c**). Similar results were also obtained when H1299 cells were treated with C225-NP in the presence of Clone 225 antibody (**Figure S4 a–c**). Taken together, these results demonstrated that C225-NP interacts selectively with EGFR-expressing NSCLC cells, and produce molecular specific enhanced therapeutic effect on EGFR-expressing NSCLC cells.

### Density of 225 antibody attached to NP is important for 225-NP-mediated tumor cell killing

To investigate the dependence of therapeutic efficacy of NPs on the density of Clone 225 antibodies on the NP surface we co-conjugated Clone 225 and non-specific IgG antibody at ratios of 1∶0, 1∶1, 1∶3, 1∶10, 1∶40 and 0∶1. The average total amount of antibodies per NP was kept the same, therefore, the density of Clone 225 antibodies gradually decreased as the relative amount of the non-specific IgG increased. For example, the ratio 1∶0 corresponded to the original C225-NP conjugates which had the highest density of Clone 225 antibodies while the ratio 0∶1 corresponded to NPs with only non-specific IgG antibodies. The relative density of antibodies on the surface of NPs was confirmed using a fluorescent assay (see Materials and Methods).

**Figure 10:**
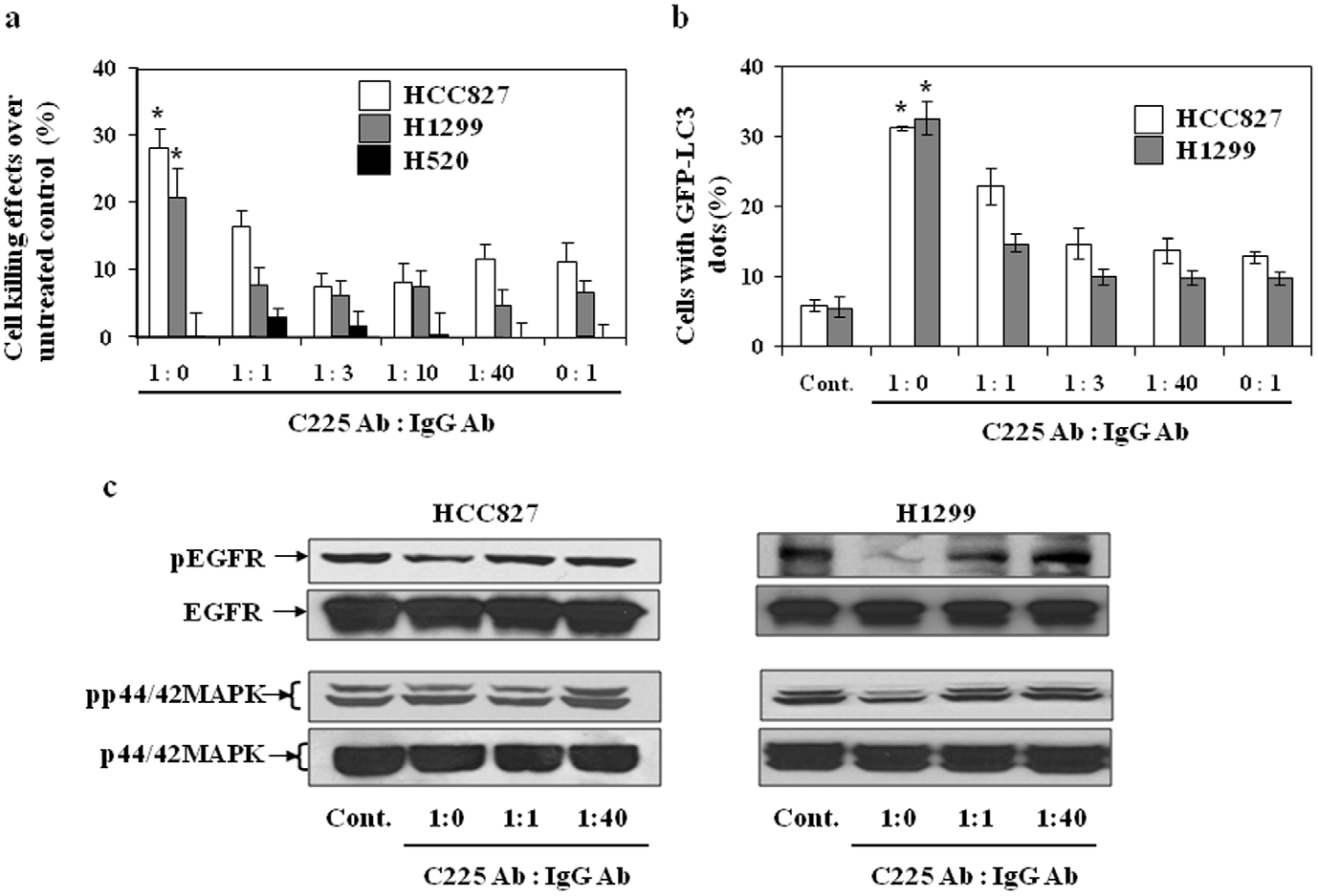
Effect of antibody density on C225-NP-mediated killing of NSCLC cells. (**a**) Cell growth inhibition in response to treatment with NP that had varying ratio (1∶0; 1∶1; 1∶3; 1∶10; 1∶40; 0∶1) of C225 and IgG co-conjugated to the NP surface. Cells (HCC827, H1299 and H520) were treated with NPs (0.6×10^10^ particles) for 72 hrs. The number of viable cells was counted as described in Materials and Methods. Results shown are the means ± S.D. of three independent experiments. *P-value <0.05 for C225-NP (1∶0) vs NP's with 1∶1–1∶40 and 0∶1 NP on HCC827 and H1299 cells. H520 cells showed no inhibitory effect when treated with NP's of different ratios. (**b**) The number of GFP-LC3 dots induced by C225-NP (1∶0)-treatment on HCC827 and H1299 cells decreased with changes in C225∶IgG antibody ratio. Results shown are the means ± S.D. of three independent experiments. *P-value <0.05 for C225-NP (1∶0) vs 1∶1–1∶40 and 0∶1 NP on HCC827 and H1299 cells. (**c**) C225-NP (1∶0)-mediated reduction in the phosphorylated EGFR and p44/42MAPK expression was abrogated in HCC827 and H1299 cells when treated with NP's of 1∶1 and 1∶40 ratio.

Treatment with C225-NPs (1∶0 ratio) significantly (*P*<0.05) decreased viability of EGFR-expressed HCC827 and H1299 cells. However, by changing the ratio of C225 antibody to control antibody from 1∶0 to 1∶40 resulted in attenuation of the cytotoxicity on both cell lines (**Figure 10a**). Ability to inhibit pEGFR expression and induce autophagy was also reduced in HCC827 and H1299 cells with decrease in the relative amount of Clone 225 antibodies on the NP surface (**Figure 10b, c**). The observed decrease in the therapeutic efficacy of the NP-antibody complex with decreasing density of the therapeutic antibody on the NP surface can be attributed to changes in multivalency of the antibody/NP complex. Previous literature reports have shown that increasing the valency of a macromolecule can result in increased binding affinity to target molecules [25], [26]. This multivalent enhancement effect was exploited to increase the efficacy of toxin inhibition through synthesis of polyvalent inhibitors [27], [28]. It was also shown that an optimal density of targeting ligands on the surface of polymers can increase internalization of ligands into cells [29]. H520 cells showed little to no cytotoxicity when treated with NPs with varying ratios of antibodies (data not shown).

These results demonstrate that the NPs produced the greatest therapeutic effect when they were coated only with the C225 antibody. Replacing the number of C225 antibody with non-specific control antibody on the surface of NP resulted in reduced anticancer activity. Additionally, this study also demonstrates the specificity of 225-NP-mediated tumor cell killing.

In conclusion, we demonstrated that EGFR-targeted C225-NPs are selectively taken up by EGFR-expressing NSCLC cells and produced enhanced antitumor activity by inducing both apoptosis and autophagy. The enhanced tumor cell killing activity produced by C225-NP is attributed to unique properties conferred by the conjugation of Clone 225 antibody with AuFe NP. To our knowledge we have demonstrated for the first time that conjugation of plasmonic nanomaterials with biologics such as Clone 225 results in synergistic antitumor properties accompanied by induction of autophagy and apoptosis. This research opens a new paradigm in development of therapeutic agents that is directed toward the ability to control the nanoscale arrangement of multiple Abs on the particle surface for enhanced targeting and therapy.

Recent preclinical studies showed that combining EGFR-targeted drugs with chemotherapy, radiation or biologics resulted in enhanced activity, suggesting combination therapy may be more useful than monotherapy [30], [31]. Therefore, we will further assess the efficiency and efficacy of these therapies in combination with C225-NPs in the future.

## Materials and Methods

### Cell culture

Human NSCLC cells (H1299, H1819, H1975, H3255, HCC827) were a generous gift from Dr. John D. Minna (Department of Internal Medicine and Pharmacology, Hammon Center for Therapeutic Oncology Research, The University of Texas Southwestern Medical Center, Dallas, Texas). NSCLC cell (H520), lung fibroblasts (MRC-9 and WI38), and normal human bronchial epithelial (NHBE) cells were purchased from American Type Culture Collection (Manassas, VA). All cells were cultured in RPMI 1640 medium (GIBCO, Grand Island, NY) or MEM Alpha (GIBCO) supplemented with 10% FCS (Hyclone, Logan, UT), 2 mM L-glutamine, penicillin (50 U/ml), and streptomycin (100 µg/ml).

### Synthesis and conjugation of nanoparticles

The protocol for preparing colloidal core/shell iron oxide/gold NPs has been previously described [32]. First, iron seeds were synthesized by coprecipitation of Fe(II) and Fe(III) chlorides (1∶2 ratio) with 1.5 M NaOH as the reductant. The colloid was magnetically decanted, washed in DIUF water (18 MOhms) via centrifugation for 2 hours at 1500×g, and resuspended in a stabilizer - 0.1 M tetramethylammonium hydroxide (TMAOH). The resulting colloidal suspension of 9 nm magnetite (Fe_3_O_4_) NPs was oxidized in boiling 0.1 M HNO_3_, followed by washing in 0.01 M HNO_3_ and resuspension in 0.1 M TMAOH. The final colloidal suspension had ∼36 mM, γFe_2_O_3_ at pH 12 with a particle diameter of *ca.* 9 nm. N(CH3)4+ and OH− ions stabilized the supension, preventing aggregation. A gold coating was formed by reducing HAuCl_4_ on the surface of the iron using an iterative hydroxylamine process under UV-Vis spectroscopy monitoring that showed a gradual increase in the plasmon resonance peak characteristic for spherical gold NPs.

The antibody conjugation procedure was followed as previously described [33]. Briefly, monoclonal antibodies were attached to the gold surface via a linker (SensoPath Technologies) that consisted of a short polyethylene glycol (PEG) chain terminated at one end by a hydrazide moiety, and at the other end by two thiol groups. First, antibodies at a concentration of 1 mg/mL were exposed to 10 mM NaIO4 in a 40 mM HEPES pH 7.4 solution for 30–40 minutes at room temperature, thereby oxidizing the hydroxyl moieties on the antibodies' Fc region to aldehyde groups. The formation of the aldehyde groups was colorimetrically confirmed using a standard assay with an alkaline Purpald solution. Then, excess hydrazide-PEG-thiol linker was added to the oxidized antibodies and was allowed to react for 20 minutes. The hydrazide portion of the PEG linker interacts with aldehyde groups on the antibodies to form a stable linkage. In this procedure a potential loss of antibody function is avoided because the linker cannot interact with the antibody's target-binding region, which contains no glycosylation. The unreacted linker was removed by a 100,000 MWCO centrifugal filter (Millipore). After purification, the modified antibodies were mixed with nanoparticles in 40 mM HEPES (pH 7.4) for 20 minutes at room temperature. During this step a stable bond is formed between the gold surface and the linker's thiol groups. Subsequently, 5% by volume of 10^−5^ M 5 kD methoxyPEG-SH (Creative PEGWorks) in water was mixed for 5 min with the antibody/NP conjugates to cap remaining bare surfaces of NPs. Then, 2% by weight polyethylene glycol (PEG) (Sigma-Aldrich) solution was added to the mixture and the colloidal suspension was washed twice at 1500×g for 20 minutes. Final conjugates were resuspended in 2% PEG in PBS.

The NPs with and without conjugated antibodies were characterized for size and charge using transmittance electron microscopy (TEM), dynamic light scattering (DLS) and Z-potential measurements. TEM analyses showed that the gold coated iron oxide NPs have sizes of 51±13 nm before and 54±11 nm after antibody conjugation (**Figure S5**). No apparent change in the sizes of NPs in TEM images is expected as antibody coating does not result in an electron dense layer that would be visible in electron microscopy. DLS measurements showed increase in NP sizes from 51±25 nm to 73±35 nm after conjugation of antibodies; this result is consistent with our previous measurements of NP/antibody conjugates [34]. Antibody conjugation also resulted in increase of surface charge from −53±3 mV to −29±1 mV as antibody and PEG molecules replace citrate ions from the surface of the citrate stabilized iron oxide core/gold shell NPs.

NPs were conjugated with anti-EGFR antibody [Clone 225 antibody, (host mouse; Sigma-Aldrich, St. Louis, MO)], anti-rabbit Ig monoclonal antibody (Clone RG-16, Sigma-Aldrich, St. Louis, MO) or with a mixture of the two antibodies at ratios of anti-EGFR∶anti-rabbit Abs of 1∶0, 1∶1, 1∶3, 1∶10, 1∶40 and 0∶1. To achieve different antibody ratios at the surface of NPs, antibodies were pre-mixed at the desired ratio before adding to NP suspension. The composition of clone 225 and anti-rabbit IgG Abs on the surface of NPs was verified using a fluorescent assay. Clone 225 Abs and anti-rabbit Abs were labeled using Alexa Fluor 488 and Alexa Fluor 594, respectively. Then calibration curves were measured to determine the concentration of labeled antibodies using fluorescent intensity. The fluorescent intensities of antibody solution at the excitation/emission wavelengths of the two Alexa fluorophores were measured at the concentration that was used for conjugation with NPs and were compared with fluorescence intensities of the supernatant after NP/antibodies conjugates were spun down. The ratio of the difference in signal between the pre- and post-conjugation intensities was calculated and was used to confirm the composition of both Abs on the surface of NPs.

### Cell viability assay

The cytotoxic effect of C225-NP on NSCLCs and normal cells was determined by using a trypan blue dye exclusion assay [35]. Cancer or normal cells were seeded at 1 or 2×10^5^ cells/well in 6-well plates and incubated overnight at 37°C. The cells were then incubated for 72 hrs with/without IgG-NP (6×10^9^ particles), C225-NP (6×10^9^ particles), or C225 antibody (0.065 ug/ml). After the cells were collected by trypsinization, they were stained with trypan blue, and the viable cells in each well were counted. The viability of the untreated cells (the control) was considered 100%. Survival fractions were calculated from the mean cell viability of the treated cells over control and the relative difference in cell number expressed as percent of cell killing over control.

### Apoptosis detection assays

Tumor cells that were untreated or treated with C225 antibody, IgG-NP or C225-NP were collected at 72 h after treatment, fixed with 70% ethanol, and stained with 10% propidium iodide solution (Roche cellular DNA flow-cytometric analysis reagent set: Indianapolis, IN) according to the manufacturer's instructions. DNA content was analyzed with a FACScan flow cytometer (Becton Dickinson) as previously described [35], and data were analyzed with the manufacturer's CellQuest software.

### Autophagy detection assay

Using the GFP-LC3 expression vector (LC3 cDNA kindly provided by Dr. N. Mizushima (Department of Physiology and Cell Biology, Tokyo Medical and Dental University), the involvement of LC3 in tumor cells treated with control IgG-NP or C225-NP was determined as previously described [36]. Tumor cells were transfected with the GFP-LC3 expression vector using FuGENE 6 transfection reagent (Roche Applied Science) or DOTAP Liposomal Transfection Reagent (Roche Applied Science). After overnight culture, cells were treated with IgG- or C225-NP fixed with 4% paraformaldehyde, and examined under an Axioskop 40 fluorescence microscope. To quantify autophagic cells after treatment, we counted the number of autophagic cells among 100 GFP-positive cells.

### Cell cycle analysis

HCC827 cells were treated with three different concentrations (1.5, 2.44 and 3.55 ng/ml) of free C225 antibody or C225-NP (0.6, 1.2, and 2.4×10^10^ particles). The amount of free C225 antibody added was at equimolar concentrations with antibodies attached to NP. Untreated cells served as controls. At 72 hrs after treatment cells were harvested and the number of cells in the sub-G_1_ phase of the cell cycle was determined by DNA flow-cytometric analysis [35].

### Western blotting

Untreated or treated cells were lysed in extraction buffer and the soluble proteins were isolated as previously described [37]. Protein concentrations were estimated using a protein assay (Bio-Rad, Richmond, CA), and proteins were separated by sodium dodecyl sulfate-7.5 to 15% polyacrylamide gel electrophoresis (SDS-PAGE) and transferred electrophoretically to Hybond-P membrane (Amersham, Piscataway, NJ). The membrane was subjected to Western blotting using anti-LC3B antibody (1∶5,000 dilution), anti-PARP antibody (1∶1000 dilution; Cell Signaling Technology, Beverly, MA), anti-phospho/total-EGFR antibody (1∶500 dilution; Santa Cruz Biotechnology, Santa Cruz, CA), anti-phosphorylated/total-AKT, p38 MAPK, and p44/42 MAPK antibodies (1∶1000 dilution; Cell Signaling Technology), and mouse anti-β-actin antibody (1∶500 dilution; Sigma-Aldrich). Anti-LC3 antibody was generated as previously described [38]. Immunoreactive proteins were detected using an enhanced chemiluminescence reagent (Amersham) according to the manufacturer's instructions.

### Electron microscopy

HCC827 cells were seeded onto 6-well plates (1×10^5^ cells/well) and were treated with C225 antibody for 72 hrs, IgG-NP or C225-NP (6×10^9^ particles) for 48 or 72 hrs and then fixed with a solution containing 3% glutaraldehyde plus 2% paraformaldehyde in 0.1 M cacodylate buffer (PH 7.3) for 1 hr. After fixation, the samples were washed and treated with 0.1% Millipore-filtered cacodylate buffered tannic acid, postfixed with 1% buffered osmium tetroxide for 30 min, and stained en bloc with 1% Millipore-filtered uranyl acetate. The samples were dehydrated in increasing concentrations of ethanol, infiltrated, and then embedded in Poly-bed 812 medium. The samples were polymerized in a 60°C oven for 2 days. Ultrathin sections were cut in Leica Ultracut microtome (Leica, Deerfield, IL), stained with uranyl acetate and lead citrate in Leica EM Stainer, and examined with a JEM 1010 transmission electron microscope (JEOL, USA, Inc., Peabody, MA) at an accelerating voltage of 80 kV. Digital images were obtained using AMT imaging system (Advanced Microscopy Techniques Corp., Danvers, MA). To quantify autophagic cells after treatment, we counted the number of autophagic cells per 100 mm^2^ cytoplasm, as previously described [39].

### Optical imaging

NSCLC and normal cells were resuspended in phenol-free RPMI medium and were seeded in two-well chamber slides. These cells were treated with IgG-NP or C225-NP in the absence or presence of free unbound C225 antibody (2 µg/ml). At 24 hrs after treatment, cells were washed, fixed in 1% paraformaldehyde, and imaged using dark-field reflectance microscopy. All dark-field images were acquired using Leica, DM6000 microscope equipped with 20× dark-field objective and Xenon-lamp white light illumination.

Confocal images were acquired with a Leica SP2 AOBS confocal microscope using a 63× oil immersion objective, 1.4 NA (Leica Microsystems, Bannockburn, IL). Images were taken every 0.25 um with a total depth of 55 um. A 633 nm red HeNe laser was used for detection of nanoparticles in reflectance mode and a UV 350 nm laser excitation was used to measure DAPI fluorescence. A 3D rendering of the confocal images was obtained using the volume rendering program Voxx (Indiana Center for Biological Microscopy).

### Immunohistochemistry for EGFR expression

HCC827 cells (2×10^3^) seeded in two-well chamber slides were treated with IgG-NP or C225-NP (0.2×10^10^ particles). At 30 minutes after treatment, chamber slides were washed in phosphate buffered saline (PBS), fixed in 0.1% glutaraldehyde and subjected to immunohistochemical staining using monoclonal antibodies against human phosphorylated and total EGFR (Santa Cruz Biotechnology) and Vectastain kit (Vector laboratories, Burlingame, CA). Untreated cells served as controls. The expression of phosphorylated and total EGFR in cells after NP treatment was captured by bright-filed microscope and reduction in phosphorylated EGFR determined by semi-quantitative analysis.

### Statistical analysis

Data are expressed as means and 95% CI. The statistical significance of the differences in the *in vitro* antitumor effects of C225 antibody, IgG-NP and C225-NP was determined by using Student's two-tailed *t* test.

## Supporting Information

Figure S1 **C225-NP reduces phosphorylated EGFR expression in NSCLC.** Ig-NP- or C225-NP-treated cells were stained for EGFR by immunocytochemistry. Phosphorylated EGFR expression was reduced in both IgG-NP and C225-NP-treated HCC827 cells when compared to untreated control cells. However, the reduction in phosphorylated EGFR expression was significantly greater in C225-NP-treated cells (35% reduction over untreated control cells; P-value <0.05) than in IgG-NP-treated cells (8% reduction over untreated control cells). Error bars denote standard deviation.
(TIF)

Figure S2 **Comparison of free C225 antibody with C225-NP on their ability to induce apoptosis in lung tumor cells.** C225-NP-treatment of HCC827 cells resulted in a dose-dependent increase in the percentage of cells in the subG1 phase compared to treatment with C225 antibody at all concentrations tested. *P-value <0.05 vs same concentrations of C225 antibody.
(TIF)

Figure S3 **C225-NP induces autophagy in H1299 lung cancer cells.** (**a**) Detection of GFP-LC3 dots in H1299 cells that were either not treated or treated with C225 antibody, IgG-NP or C225-NP (3×10^9^ particles) for 72 hrs on chamber slides. *Scale bar = 50 µm* (**b**) Quantitative analysis showed C225-NP-treated HCC827 cells had higher number of GFP-LC3 dots in compared to all other treatment groups. Results shown are the means ± S.D. of three independent experiments. *P-value <0.05 vs untreated control, C225 antibody, and IgG-NP.
(TIF)

Figure S4 **Visualization, and determination of selective binding and uptake of C225-NP in H1299 cells.** (**a**) In the right column cells were treated with C225 antibody (2 µg/ml) for 15 min, and then incubated with either IgG-NP or C225-NP for additional 24 hrs. The left column shows cells which were not pre-treated with free antibodies. The slides were washed, fixed and imaged under dark-field microscopy. Binding and uptake of C225-NP was completely inhibited in the presence of C225 antibody. In IgG-NP-treated cells C225 antibody had no effect. Scale bar is 50 micron. (**b**) Inhibition effects of free C225 antibody on the cytotoxicity of C225-NP by pre-treatment with free C225 antibody. After treatment with C225 antibody (0.065 µg/ml) for 6 hrs, the cells were treated with C225-NP for an additional 66 hrs. Cells treated for 66 hrs with C225-NP, C225 antibodies alone, non-conjugated NP (gold-iron: AuFe), and IgG-NP were used for comparison. Results shown are the means ± S.D. of three independent experiments. *P-value <0.05 vs AuFe alone, C225 antibody alone, IgG-NP or C225 antibody plus C225-NP. (**c**) Inhibition effects of pre-treatment with C225 antibody on C225-NP-induced apoptosis and autophagy. Cellular proteins were lysed after treatment with C225-NP (0.6×10^10^ particles) for 66 hrs in the presence or absence of free C225 antibody (0.065 µg/ml). Proteins were separated by 7.5% or 15% SDS-PAGE, and immunoblotted with anti-PARP and anti-LC3 antibodies. The intensities of the amount of LC3-II bands were quantified by ImageJ software (National Institutes of Health). C225-NP-mediated activation of apoptosis and autophagy as indicated by cleavage of capase-3 and LC3-II respectively were markedly abrogated in the presence of free C225 antibody. PARP cleavage was not detectable in all of the groups.
(TIF)

Figure S5 **Nanoparticle size determination by transmission electron microscopy.** Size analyses at lower and higher magnification showed antibody-conjugated NPs were 54±11 nm in size. *Scale bar = 500 and 50 nm.*
(TIF)
