# Supplementary figures and images for: EGFR-Targeted Hybrid Plasmonic Magnetic Nanoparticles Synergistically Induce Autophagy and Apoptosis in Non-Small Cell Lung Cancer Cells
Source: PLoS One. 2011 Nov 7;6(11):e25507. doi: 10.1371/journal.pone.0025507 (PMC3210119; doi:10.1371/journal.pone.0025507)

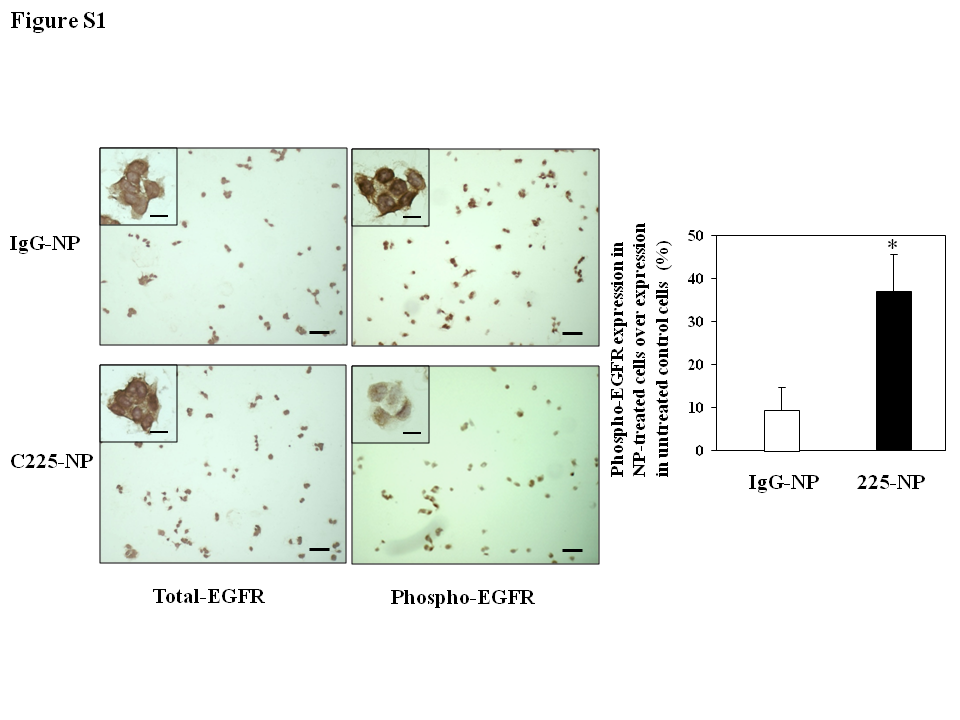

Supplement: Figure S1 — C225-NP reduces phosphorylated EGFR expression in NSCLC. Ig-NP- or C225-NP-treated cells were stained for EGFR by immunocytochemistry. Phosphorylated EGFR expression was reduced in both IgG-NP and C225-NP-treated HCC827 cells when compared to untreated control cells. However, the reduction in phosphorylated EGFR expression was significantly greater in C225-NP-treated cells (35% reduction over untreated control cells; P-value <0.05) than in IgG-NP-treated cells (8% reduction over untreated control cells). Error bars denote standard deviation. (TIF) [file pone.0025507.s001.tif]

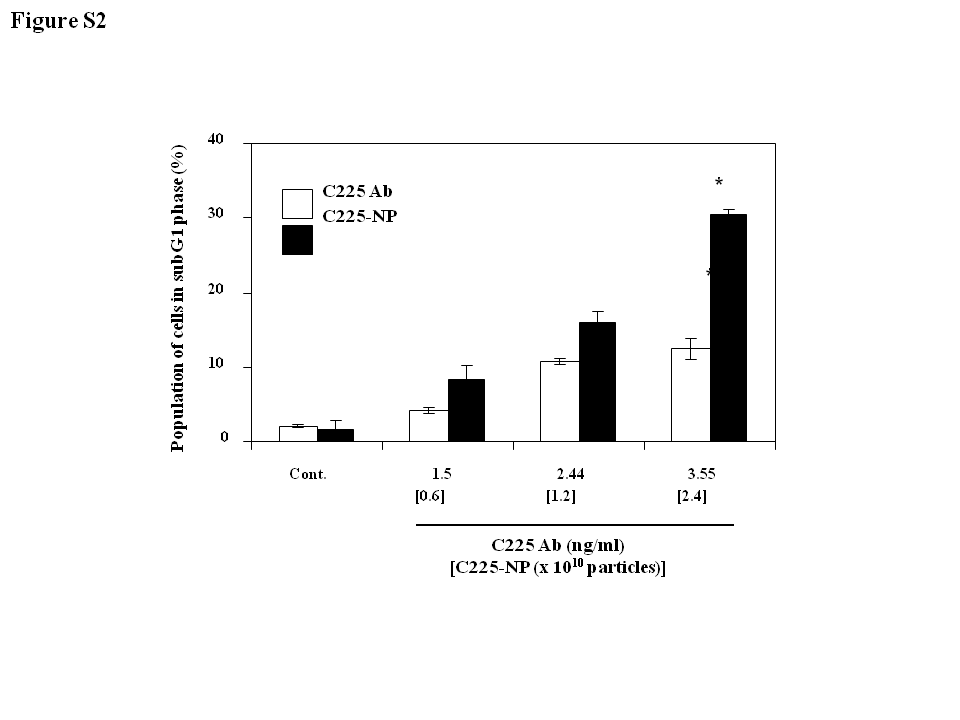

Supplement: Figure S2 — Comparison of free C225 antibody with C225-NP on their ability to induce apoptosis in lung tumor cells. C225-NP-treatment of HCC827 cells resulted in a dose-dependent increase in the percentage of cells in the subG1 phase compared to treatment with C225 antibody at all concentrations tested. *P-value <0.05 vs same concentrations of C225 antibody. (TIF) [file pone.0025507.s002.tif]

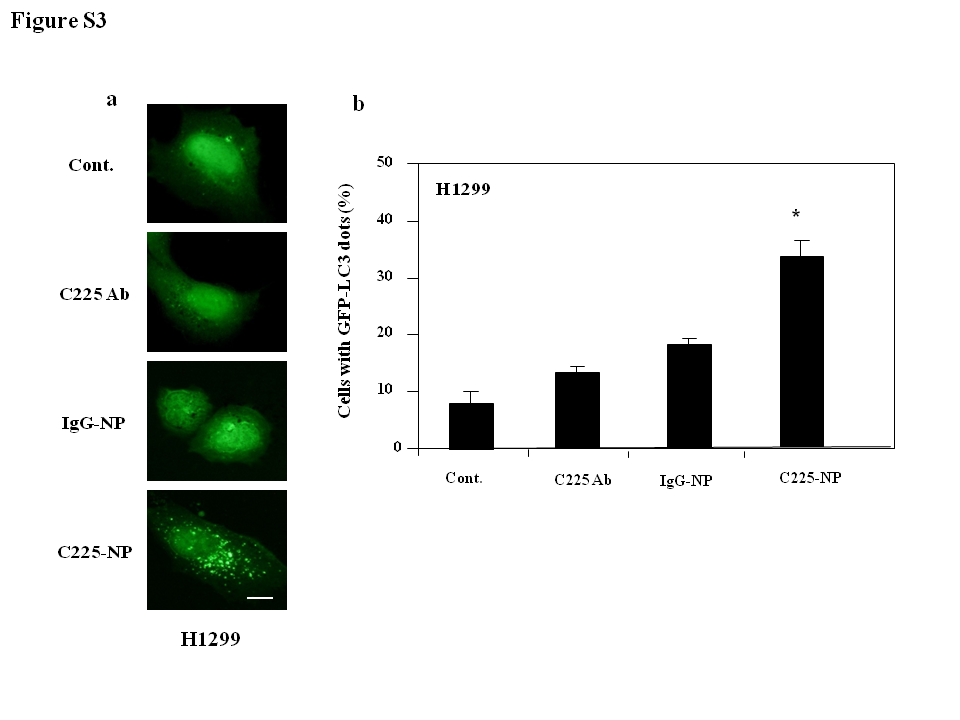

Supplement: Figure S3 — C225-NP induces autophagy in H1299 lung cancer cells. (a) Detection of GFP-LC3 dots in H1299 cells that were either not treated or treated with C225 antibody, IgG-NP or C225-NP (3×10^9^ particles) for 72 hrs on chamber slides. Scale bar = 50 µm (b) Quantitative analysis showed C225-NP-treated HCC827 cells had higher number of GFP-LC3 dots in compared to all other treatment groups. Results shown are the means ± S.D. of three independent experiments. *P-value <0.05 vs untreated control, C225 antibody, and IgG-NP. (TIF) [file pone.0025507.s003.tif]

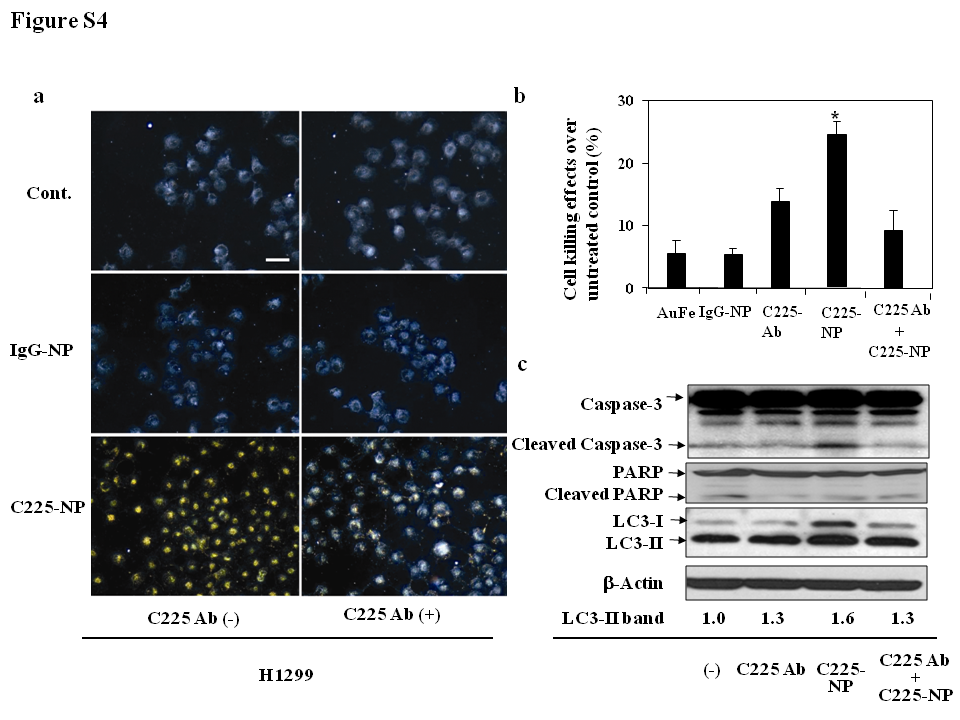

Supplement: Figure S4 — Visualization, and determination of selective binding and uptake of C225-NP in H1299 cells. (a) In the right column cells were treated with C225 antibody (2 µg/ml) for 15 min, and then incubated with either IgG-NP or C225-NP for additional 24 hrs. The left column shows cells which were not pre-treated with free antibodies. The slides were washed, fixed and imaged under dark-field microscopy. Binding and uptake of C225-NP was completely inhibited in the presence of C225 antibody. In IgG-NP-treated cells C225 antibody had no effect. Scale bar is 50 micron. (b) Inhibition effects of free C225 antibody on the cytotoxicity of C225-NP by pre-treatment with free C225 antibody. After treatment with C225 antibody (0.065 µg/ml) for 6 hrs, the cells were treated with C225-NP for an additional 66 hrs. Cells treated for 66 hrs with C225-NP, C225 antibodies alone, non-conjugated NP (gold-iron: AuFe), and IgG-NP were used for comparison. Results shown are the means ± S.D. of three independent experiments. *P-value <0.05 vs AuFe alone, C225 antibody alone, IgG-NP or C225 antibody plus C225-NP. (c) Inhibition effects of pre-treatment with C225 antibody on C225-NP-induced apoptosis and autophagy. Cellular proteins were lysed after treatment with C225-NP (0.6×10^10^ particles) for 66 hrs in the presence or absence of free C225 antibody (0.065 µg/ml). Proteins were separated by 7.5% or 15% SDS-PAGE, and immunoblotted with anti-PARP and anti-LC3 antibodies. The intensities of the amount of LC3-II bands were quantified by ImageJ software (National Institutes of Health). C225-NP-mediated activation of apoptosis and autophagy as indicated by cleavage of capase-3 and LC3-II respectively were markedly abrogated in the presence of free C225 antibody. PARP cleavage was not detectable in all of the groups. (TIF) [file pone.0025507.s004.tif]

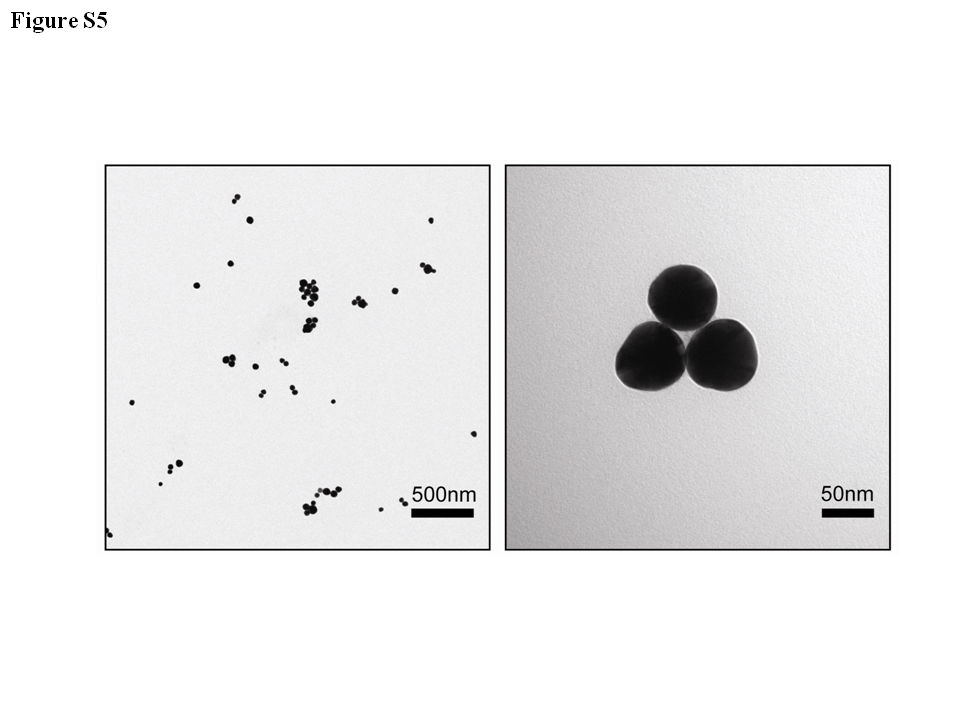

Supplement: Figure S5 — Nanoparticle size determination by transmission electron microscopy. Size analyses at lower and higher magnification showed antibody-conjugated NPs were 54±11 nm in size. Scale bar = 500 and 50 nm. (TIF) [file pone.0025507.s005.tif]
